# Implications of silver nanoparticles for *H. pylori* infection: modulation of CagA function and signaling

**DOI:** 10.3389/fcimb.2024.1419568

**Published:** 2024-06-25

**Authors:** Lucie Hochvaldova, Gernot Posselt, Silja Wessler, Libor Kvítek, Aleš Panáček

**Affiliations:** ^1^ Department of Physical Chemistry, Palacky University Olomouc, Olomouc, Czechia; ^2^ Department of Biosciences and Medical Biology, Paris Lodron University of Salzburg, Salzburg, Austria; ^3^ Cancer Cluster Salzburg (CCS), Salzburg, Austria; ^4^ Center for Tumor Biology and Immunology (CTBI), Paris Lodron University of Salzburg, Salzburg, Austria

**Keywords:** silver nanoparticles, *H. pylori*, AGS, infection, IL-8, CagA, minimum inhibitory concentration

## Abstract

**Background:**

Helicobacter pylori infection poses a significant health burden worldwide, and its virulence factor CagA plays a pivotal role in its pathogenesis.

**Methods:**

In this study, the interaction between H. pylori-infected AGS cells and silver nanoparticles (AgNPs) was investigated, with a focus on the modulation of CagA-mediated responses, investigated by western blotting. Both, the dose-dependent efficacy against H. pylori (growth curves, CFU assay) and the impact of the nanoparticles on AGS cells (MTT assay) were elucidated.

**Results:**

AGS cells infected with H. pylori displayed dramatic morphological changes, characterized by elongation and a migratory phenotype, attributed to CagA activity. Preincubation of H. pylori with AgNPs affected these morphological changes in a concentration-dependent manner, suggesting a correlation between AgNPs concentration and CagA function.

**Conclusion:**

Our study highlights the nuanced interplay between host-pathogen interactions and the therapeutic potential of AgNPs in combating H. pylori infection and offers valuable insights into the multifaceted dynamics of CagA mediated responses.

## 1 Introduction

In the field of antimicrobial research, silver nanoparticles (AgNPs) have attracted considerable attention due to their strong antimicrobial properties against a broad spectrum of bacterial pathogens, including multi-resistant variants. For instance, AgNPs have shown promising results against ESKAPE pathogens, which are known for their multi-drug resistance and the ability to cause nosocomial infections. These pathogens include *Enterococcus faecium, Staphylococcus aureus, Klebsiella pneumoniae, Acinetobacter baumannii, Pseudomonas aeruginosa*, and *Enterobacter* species and growth inhibition has been observed with the application of extremely low concentrations of silver nanoparticles against all these tested bacteria (Wahab et al., 2021; Mateo and Jiménez, 2022; Khan et al., 2023). AgNPs exhibit robust intrinsic antimicrobial properties, while enhancing the efficacy of antibiotics that have lost their effectiveness against resistant pathogens. This mutual interaction not only restores the therapeutic efficacy of established antibiotics but also broadens the spectrum of available treatment modalities (Lopez-Carrizales et al., 2018; Hochvaldová et al., 2022). In contrast to conventional antibiotics, AgNPs act at multiple levels (Slavin et al., 2017; Wang et al., 2017) and could represent a true alternative to them. *In vitro* studies have demonstrated AgNP-mediated inhibition of bacterial growth through various mechanisms including membrane disruption, damage of intracellular components and interference with essential cellular processes (Saravanakumar et al., 2019; Grande et al., 2020; Jang et al., 2022). Therefore, AgNPs provide a promising avenue for the development of innovative therapeutic approaches. However, research on the effect of nanoparticles on the pathogenicity-associated processes of infectious bacteria has been limited and is leaving a critical gap in understanding the potential of this alternative treatment approach.

*Helicobacter pylori* (*H. pylori*) infection is prevalent and of paramount clinical significance, affecting more than 50% of the world’s population. Persistent infection with *H. pylori* is intricately linked to chronic gastritis, peptic ulcers, and has been associated with an increased risk of developing gastric cancer, one of the leading causes of cancer-related death (Pormohammad et al., 2019). Current *H. pylori* eradication regimens predominantly rely on a combination of antibiotics and proton pump inhibitors in triple and quadruple therapy (Malfertheiner et al., 2023). While this approach has shown success rates in the range of 70% to 90%, the rise of antibiotic-resistant strains poses a considerable challenge to its effectiveness (Ding et al., 2023). As a result, researchers are actively exploring alternative strategies to combat *H. pylori* infection.

*H. pylori* pathogenicity factors directly affect the infected gastric epithelial cells. One well-characterized factor is the Cytotoxin-associated gene A (CagA), which plays a central role in *H. pylori*-driven pathogenesis and CagA positive strains are associated with an enhanced risk of developing gastric cancer (Takahashi-Kanemitsu et al., 2020; Alipour, 2021). Through a specialized type IV secretion system CagA is translocated into the cytoplasm of host cells, where it affects cancer-associated signal transduction pathways (Krisch et al., 2016). Phosphorylated CagA recruits important signalling molecules into a large multi-enzyme complex, that controls several important cell responses. Among the translocated effectors of the type-IV secretion system is the recently discovered ADP-heptose (Pfannkuch et al., 2019), which together with CagA controls NF-κB (nuclear factor kappa B)-mediated proinflammatory cytokine responses (Brandt et al., 2005; Faass et al., 2021), morphological changes (Takahashi-Kanemitsu et al., 2020), production of reactive oxygen species (Sah et al., 2023), DNA damage, and changes in the transcriptional signature (Bauer et al., 2020) leading to tissue injury and neoplastic transformation (Rizzato et al., 2020).

In recent decades, the widespread use of antibiotics for eradication of *H. pylori* with antibiotics has contributed to a massive increase in antibiotic-resistant *H. pylori*, as well as other human-pathogenic bacteria (Boyanova et al., 2023). Therefore, the development of novel strategies to combat antibiotic resistant bacteria is important. Here, we analyzed the impact of AgNPs on *H. pylori* viability and pathogenesis and investigated whether the AgNPs affect viability of gastric epithelial cells. Further we investigated whether AgNPs interfere with CagA translocation and phosphorylation. Finally, our data point to the utility of AgNPs as a novel antimicrobial agent against *H. pylori*.

## 2 Materials and methods

### 2.1 Synthesis and characterization of silver nanoparticles

Synthesis of AgNPs followed the previously published Tollens process (Panáček et al., 2006), however, in this case after [Ag(NH_3_)_2_]^+^ complex cation formation by mixing silver nitrate (p.a., Fagron), and ammonia solutions (28–30% [w/w], p.a., Sigma–Aldrich), gelatine (Sigma–Aldrich) was added before initiating the reduction process by sodium borohydride (Sigma–Aldrich) to prevent AgNPs aggregation caused by introduction to culture medium. All the constituents were added at vigorous stirring at the laboratory temperature and the concentrations were as outlined below: silver nitrate at 1·10^−3^ mol·dm^−3^ (equivalent to a mass concentration of 108 μg/ml of Ag), ammonia at 5·10^−3^ mol·dm^−3^, gelatine 0,05% and 1·10^−2^ mol·dm^−3^ of sodium borohydride.

The particle size was measured by dynamic light scattering (DLS, Malvern Zetasizer Nano Series, UK) and was confirmed by transmission electron microscopy (TEM) using the JEM 2010 instrument (Jeol, Japan). UV/VIS spectra and surface plasmon resonance of silver nanoparticles was recorded by the Specord S 600 spectrophotometer (Analytik Jena, Germany).

### 2.2 Helicobacter pylori culture

*H. pylori* wild-type strain (P12) (Poppe et al., 2007), known for its expression of Western CagA was cultured on GC agar plates supplemented with 10% horse serum (Th. Geyer, Germany) under microaerophilic conditions at 37°C for 48 hours. For determination of antimicrobial activity in liquid culture brain heart infusion (BHI, Sigma-Aldrich, Vienna) broth containing 10% fetal calf serum (FCS, Biowest, France) was used. Bacterial growth associated with optical density (OD) at 600 nm and absorption at various wavelengths was measured on CLARIOstar plate reader (BMG Labtech, Germany).

### 2.3 Real time monitoring of bacterial growth

The minimum inhibition concentration (MIC) was determined through a modified microdilution method. Serial two-fold dilutions of silver nanoparticles were prepared in water, and 100 µl of each concentration were transferred into individual wells. Subsequently, 100 µl of bacterial suspension in BHI medium (Sigma-Aldrich, Vienna) OD_600_ 0.2 was added (liquid culture with a starting OD_600_ 0.1 grown at 37°C, microaerophilic conditions, 40 rpm for 36 hours prior the experiment).The 96-well plates were then incubated at 37°C, in 10% CO_2_ and 5% O_2_ atmosphere, double orbital shaking 500 rpm for 24 hours in a plate reader (CLARIOstar, BMG Labtech, Germany), while OD at 600 nm was measured every 30 minutes.

### 2.4 CFU assay

It is noteworthy that the design of the colony-forming units (CFU) assay was purposefully tailored with infection experiments in mind. For CFU assays and infection experiments, phosphate-buffered saline (PBS, Sigma-Aldrich, Vienna)) served as the dilution medium for AgNPs. 500 µl of AgNPs dispersion at different concentrations were mixed with 10^8^ bacteria in 500 µl PBS and incubated at 37°C for 2 hours under microaerophilic conditions. 10 μl of the solution were used for the CFU assay, the rest was centrifuged and used for the infection experiments. For the CFU assay, each sample was diluted 1:10000 and aliquots of 100 µl from each dilution were plated on GC agar plates. Subsequently, the plates were incubated at 37°C for 3 days, then, the number of CFU was determined.

### 2.5 Cell culture and infection

AGS cells (a human gastric adenocarcinoma cell line, ECACC, UK) were grown in RPMI 1640 medium (Sigma-Aldrich, Germany), supplemented with 2 mM L-glutamine (Biowest, Germany), and 10% FCS (Biowest, France) within a humidified 5% CO_2_ atmosphere at 37°C. For infection experiments, AGS cells were seeded in tissue culture dishes (Greiner, Germany) 48 hours prior to infection and were allowed to grow until 70% confluence. One hour prior to infection, the medium was replaced with serum-free medium. Bacteria were collected in PBS and mixed with nanoparticles as described within CFU assay paragraph.

Where indicated two *H. pylori* controls were implemented: First, untreated bacteria were directly added to the cells (Hp0) and second, *H. pylori* was incubated in PBS for two hours (Hp2) under microaerophilic conditions prior to infection (**Supplementary Figure S1**) to control the effect of this incubation step in absence of AgNPs. As an additional control, AGS cells were incubated with the highest concentration of AgNPs (13.5 µg/ml) to determine the direct impact of AgNPs on AGS cells in the infection experiments.

Host cells were infected with a multiplicity of infection (MOI) 20 for 4 hours. In mock infected samples, an equivalent volume of PBS was added. After four hours of infection images were captured using a phase-contrast microscope (CKX41, Olympus, Austria). Cells were then carefully washed twice with ice-cold PBS and subsequently lysed in a modified RIPA buffer containing 20 mM Tris (pH 7.5), 1 mM EDTA, 100 mM NaCl, 0.5% DOC, 0.1% SDS, 1% Triton X-100, 20 mM β-glycerophosphate, 20 mM NaF, 1 mM Na_2_MoO_4_, 1 mM Na_3_VO_4_, and 1× complete protease inhibitor cocktail (all from Sigma-Aldrich, Vienna) and cleared from debris by centrifugation.

Cell elongation was quantified by counting of normal and elongated cells in the field of view (fov) for each experimental condition. Five images were randomly selected from different areas within each condition. The average and standard deviation of percent elongated cells were then calculated for each condition. Images from three independent experiments were analyzed.

### 2.6 Western blotting

30 μg of whole cell lysates were loaded separated by SDS PAGE using 8–10% polyacrylamide gels. The proteins were transferred onto nitrocellulose membranes (Carl Roth, Karlsruhe, Germany) by semidry transfer blotting (BioRad, Germany). Membranes were blocked in 3% bovine serum albumin (Carl Roth, Germany), supplemented with 0.05 mM Na_3_VO_4_ (Sigma-Aldrich, Vienna), and incubated with primary antibodies overnight at 4°C, followed by incubation with species specific HRP-coupled secondar antibodies (Thermo Fisher Scientific, Germany) for two hours at room temperature and developed using the ChemiDoc XRS+ System (Bio-Rad, Germany). Following antibodies were used: Anti-phosphotyrosine antibody 4G10 (Cell Signaling, Frankfurt am Main, Germany); anti-GAPDH (Cell Signaling, Frankfurt am Main, Germany) and polyclonal anti-CagA antibodies (Krisch et al., 2016).

### 2.7 ELISA

ELISA assay for IL-8 was conducted using the Human IL-8 (CXCL-8) Standard ELISA Development kit (Peprotech, London UK) following the manufacturer’s instructions. Duplicate analyzes were performed for samples from three distinct experiments. The standard fitting curve was generated utilizing a five-parameter logistic non-linear regression model (5-PL).

### 2.8 Cytotoxicity evaluation

Cell viability assays were conducted using 10^4^ AGS cells per well in 96-well tissue culture plates. Cells were incubated with AgNPs for 24 h in 100 µl RPMI, 2 mM L-Glu, 10% FCS. Metabolic activity was assessed as a measure of cell viability by adding 10 μl of 5 mg/ml MTT (3-(4,5-dimethylthiazol-2-yl)2 2,5-diphenyl tetrazolium bromide; (Sigma-Aldrich, Germany) to the medium for 1 hour at 37°C. The cells were then lysed using 110 μl of MTT lysis solution (0.1% NP-40, 0.04 N HCl in isopropanol), and the absorbance was measured at 570 nm using a plate reader (M200 Pro, Tecan, Austria). Cell survival for treated cells was normalized to the levels of their respective non-treated controls. The results were based on the mean of three independent experiments. The IC50 values for bacteria and cells were determined using polynomial regression analysis. R-squared value of 0.99 provided accurate representations of the dose-response relationship. Subsequently, IC50 values were extrapolated from these curves using quadratic equations derived from the trend lines.

### 2.9 Statistical analysis

Statistical analyzes were conducted in GraphPad Prism using One-way ANOVA with Dunnett’s multiple comparison procedure.

## 3 Results

### 3.1 Synthesis and characterization of silver nanoparticles

**Figure 1:**
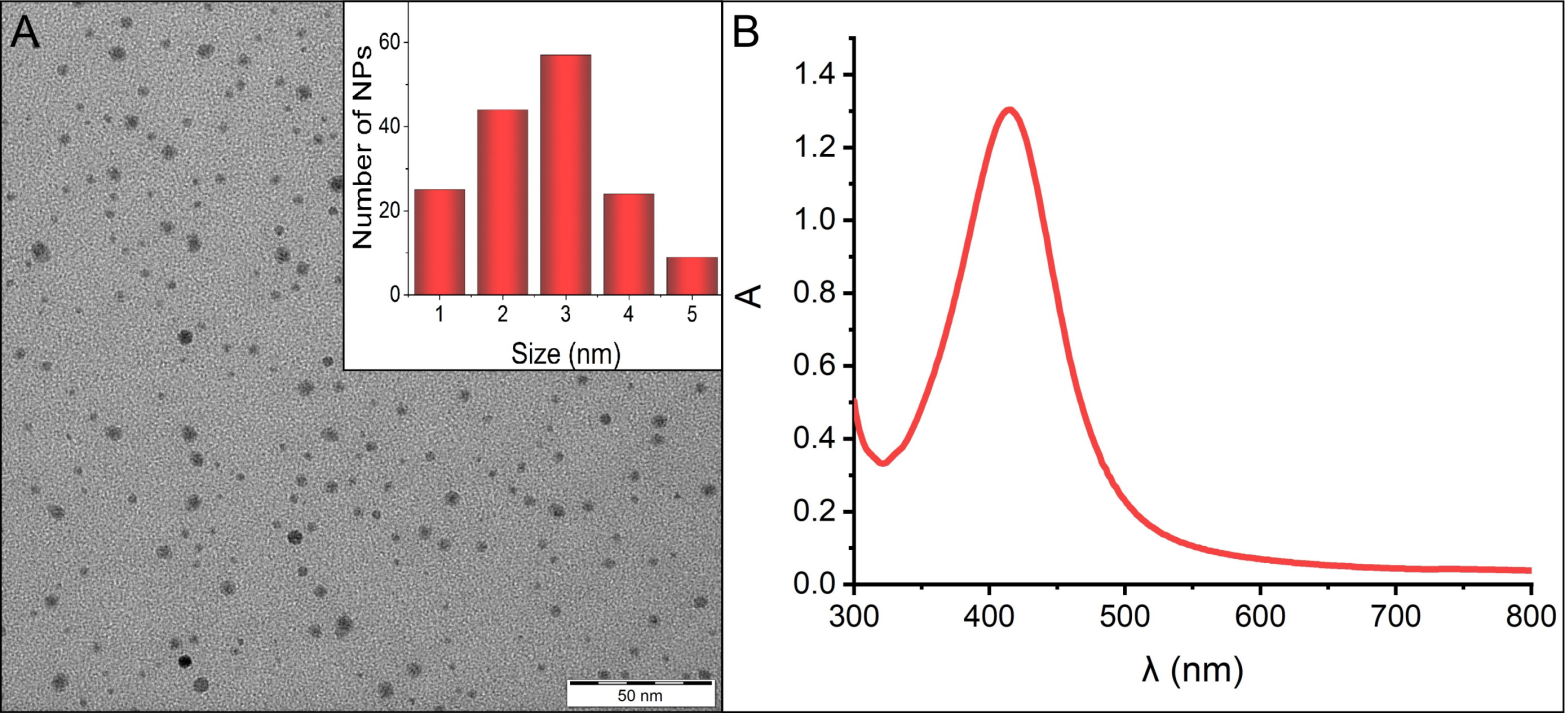
**(A)** TEM image displaying the morphology of AgNPs, along with the size distribution inset in the panel. **(B)** Absorption spectrum of AgNPs, indicating the characteristic surface plasmon resonance peak at 415 nm.

For the purposes of this study, relatively small AgNPs were synthesized utilizing a strong reducing agent (sodium borohydride). To prevent nanoparticle aggregation in the bacterial medium, gelatine was incorporated as a stabilizing agent. The mean diameter of 3 nm and the narrow size distribution of the spherical nanoparticles, as determined by DLS, were further supported by transmission electron microscopy (TEM) (**Figure 1A**). Complementing this, UV/VIS spectra, marked by distinct surface plasmon resonance at 415 nm were recorded (**Figure 1B**).

### 3.2 Antibacterial effect of AgNPs on *H. pylori* growth

**Figure 2:**
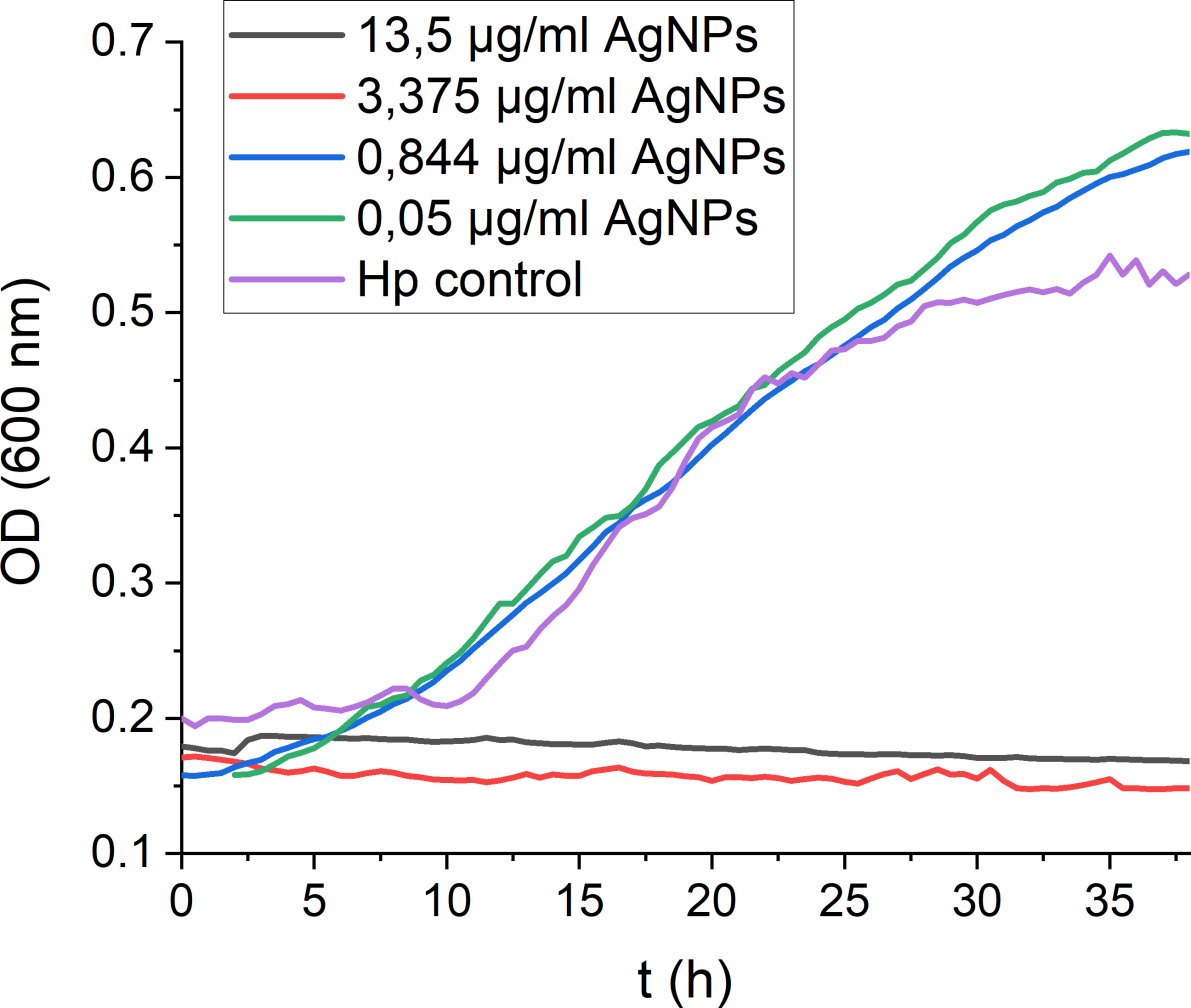
Real-time monitoring of *H. pylori* growth in the presence of decreasing AgNPs concentrations. Exemplary growth curves demonstrating the consistent trends observed across triplicate measurements.

To assess the antimicrobial potential of the AgNPs towards *H. pylori*, real-time growth monitoring of *H. pylori* cultures under microaerophilic conditions was performed. Monitoring *H. pylori* growth under exposure to different concentrations of AgNPs (**Figure 2**) revealed crucial insights into the MIC and the antimicrobial efficacy of the nanoparticles and offered a dynamic representation of the inhibitory effect over 48 hours. A strong dose-dependent inhibition of *H. pylori* growth was observed, with higher concentrations of nanoparticles leading to more pronounced growth inhibition. The point at which the growth curve significantly deviates from the growth control signifies the MIC, which is the lowest concentration (3.375 μg/ml) at which bacterial growth is effectively inhibited, suggesting that AgNPs exhibit an efficient antimicrobial activity against *H. pylori.*

**Figure 3:**
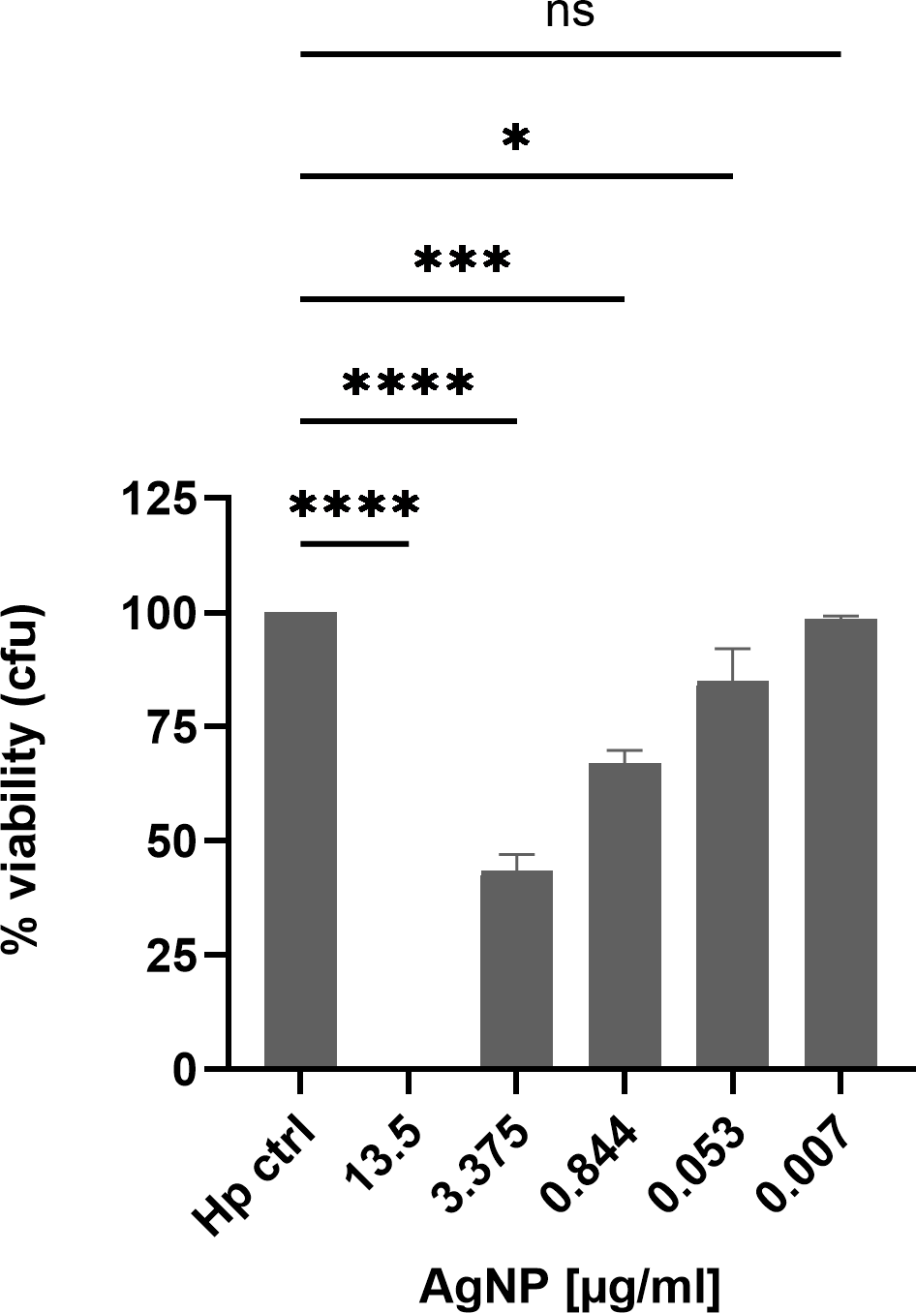
*H. pylori* viability after 2 h treatment with different concentrations of AgNPs in a CFU assay. Quantitative data are presented as mean ± standard deviation of cell viability percentages from three independent experiments. The reference “Hp” signifies the condition without nanoparticles, serving as the non-treatment control and was defined as 100%. ns: not significant; * p≤0.05 , *** p≤0.001, **** p≤0.0001.

The decrease in *H. pylori* growth could indicate both, inhibition of growth or bacterial cell death. Therefore, to quantify viable bacteria subsequent to exposure to different nanoparticle concentrations, CFU (colony forming unit) assays were performed to provide a precise quantitative assessment of bacterial survival after two hours of incubation with nanoparticles. With increasing AgNPs concentrations a strong dose-dependent reduction in CFU´s was observed (**Figure 3**), signifying a bactericidal effect of the nanoparticles. At a concentration of 3.375 μg/ml, more than 60% of the bacterial population was eliminated within a time span of two hours. An IC50 value of 2.056 μg/ml was calculated by plotting a dose-response curve. Notably, the entire bacterial population was eradicated at a concentration of 13.5 μg/ml. These findings underscore the effective antimicrobial properties of AgNPs, with MICs comparable to previously published data for *H. pylori and* other bacteria (Amin et al., 2014; Gurunathan et al., 2015; Saravanakumar et al., 2019).

### 3.3 Cytotoxic effect of AgNPs on eukaryotic host cells

**Figure 4:**
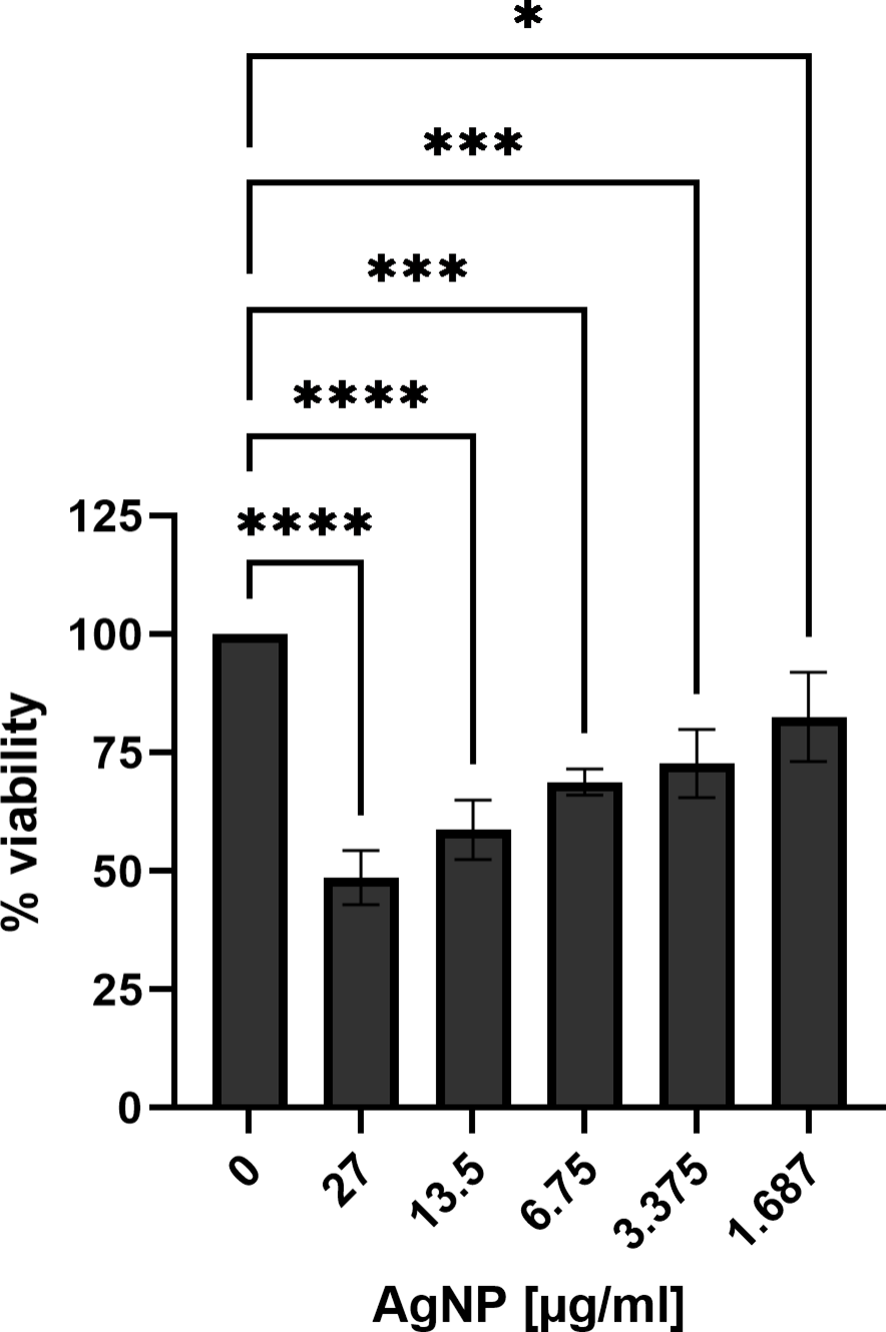
Cell viability of AGS cells treated with different concentrations of AgNPs for 24 h in RPMI supplemented with L-glutamine and FCS. Quantitative data are presented as mean ± standard deviation of cell viability percentages from three independent experiments. The reference “0” signifies the condition without nanoparticles, serving as the non-treatment control and was set to 100% viability. * p≤0.05, *** p≤0.001, **** p≤0.0001.

To assess the toxicity potential of silver nanoparticles towards eukaryotic cells, MTT assays were performed to gain insight into the biocompatibility of AgNPs with the gastric epithelial cell line AGS, a well-established model for *H. pylori* infection (Moese et al., 2004). Assessment of cell viability after incubation with different concentrations of AgNPs revealed a concentration-dependent impact on AGS cell viability (**Figure 4**). The viability for the non-treated cells was set as 100%. A significant reduction in cell viability was observed in response to increasing concentrations of AgNPs. Exposure to the highest concentration tested (27 μg/ml) resulted in a decrease of cell viability to 49%, while at 13.5 μg/ml and 6.75 μg/ml, viability decreased to 59% and 69%, respectively, compared to control cells. Moreover, at 3.375 μg/ml, viability dropped to 73%, and the lowest concentration of 1.68 μg/ml, resulted in 83% cell viability. The IC50 for AGS cells was calculated to be 24.08 μg/ml.

### 3.4 Effects of AgNPs on AGS cell morphology, CagA translocation and phosphorylation, and cytokine expression in *H. pylori* infection

**Figure 5:**
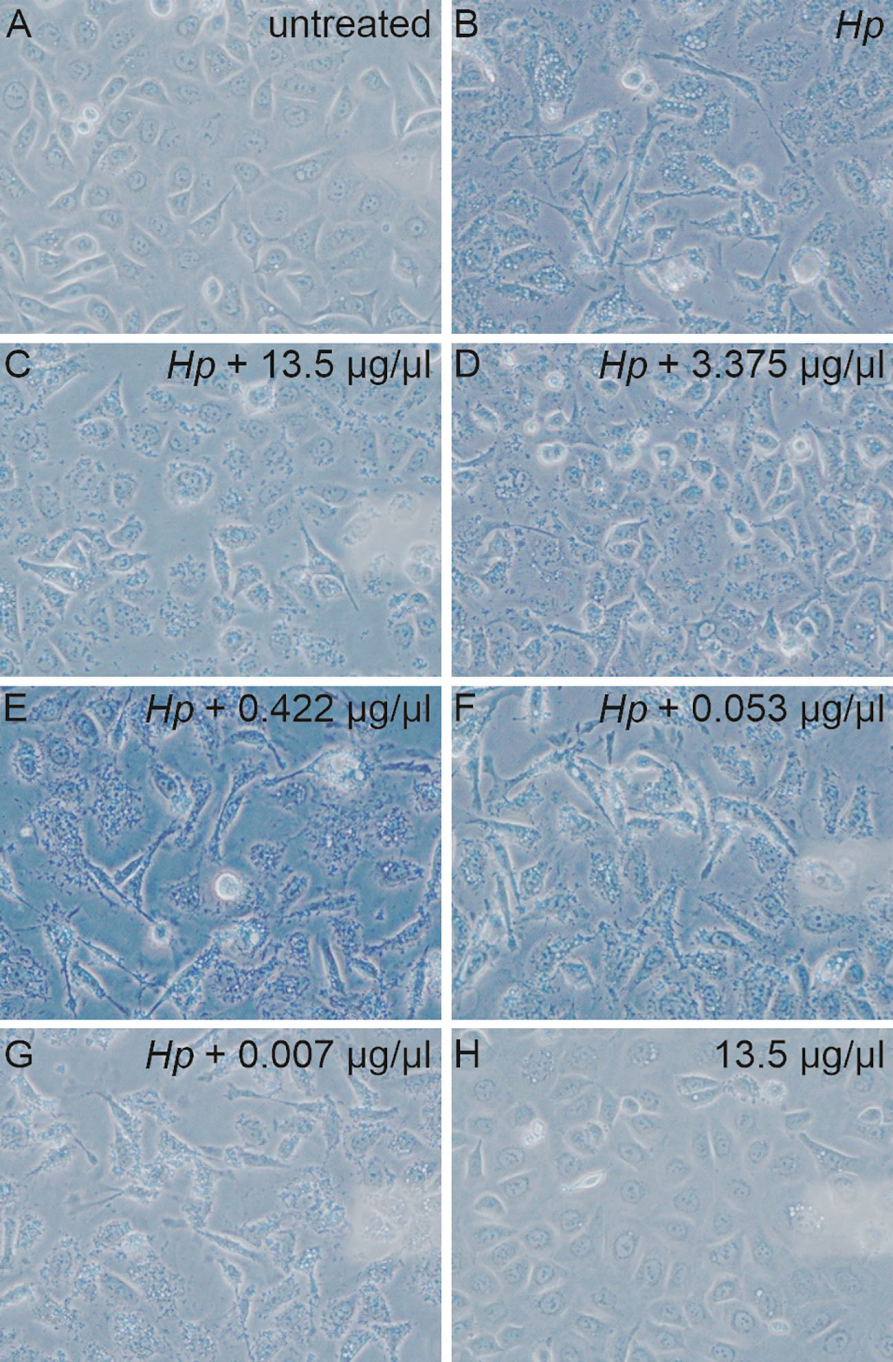
Microscopic images of the AGS cells non-infected **(A, H)** and infected with bacteria **(B)** pre-treated with various concentration of AgNPs for 2 hours **(C-G)**. 40x magnification.

**Figure 6:**
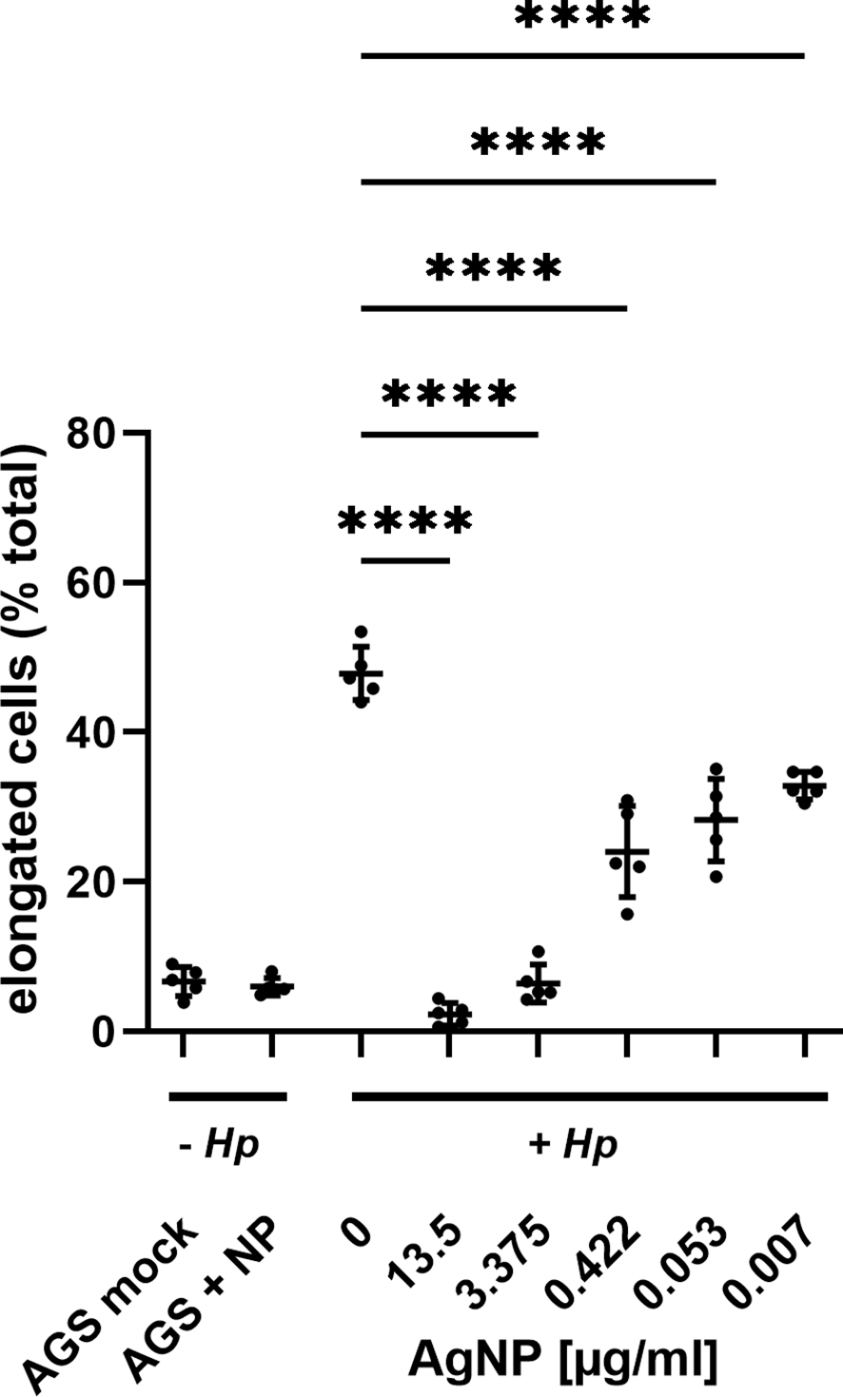
Evaluation of AGS cell elongation in response to increasing AgNPs concentration. Cell elongation was quantified by assessing the proportion of elongated cells relative to the total cell population in the field of view (fov) for each experimental condition. Images from three independent experiments were analyzed, with cell morphology assessed in 5 randomly selected samples per condition. **** p≤0.0001.

Next, the effect of AgNPs was investigated in an experimental model of *H. pylori* infection. Here, *H. pylori* was incubated with various concentrations of AgNPs for two hours prior to infection. AGS cells were then infected with *H. pylori* for four hours and the changes in cell morphology were analyzed by phase-contrast microscopy (**Figure 5**). Uninfected AGS cells and cells treated with nanoparticles alone (13.5 μg/ml) in absence of *H. pylori* show the typical epithelial cell morphology with intact cell-cell contacts. A high percentage of elongated cells was observed after infection with *H. pylori*. Pretreatment with increasing concentrations of AgNPs resulted in a strong reduction of the cell elongation phenotype in response to infection (**Figure 6**).

**Figure 7:**
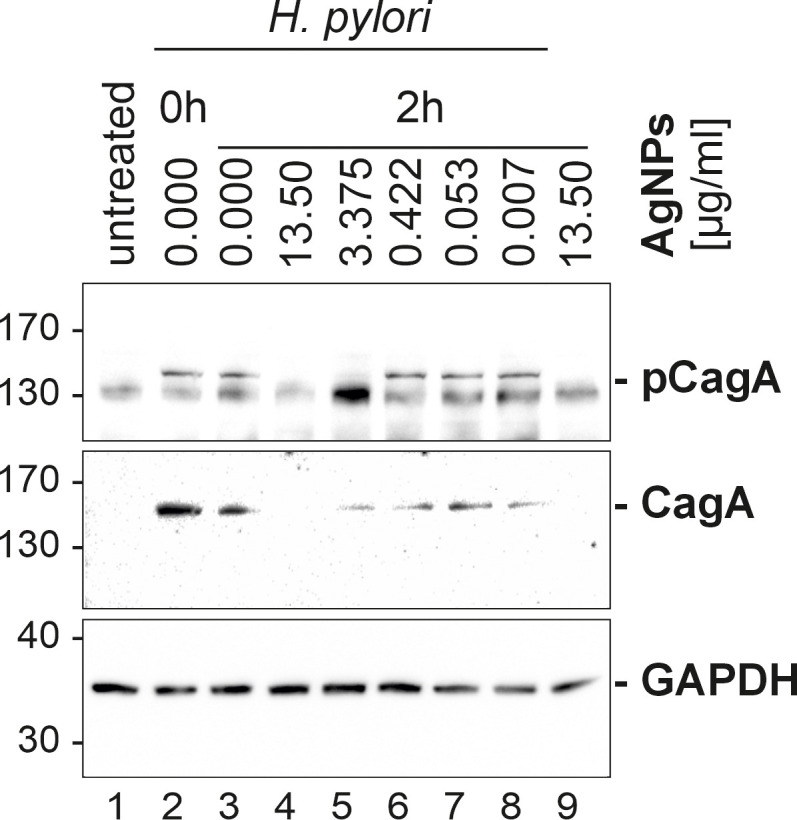
Western blot of infected AGS cells. AGS cells (samples 2–8) were infected with *H. pylori*, sample 1 served as control (AGS cells only) and sample 9 served as an AgNPs toxicity control (mock infection with the highest used AgNPs concentration, without bacteria). Sample 2 (Hp0 – immediate infection), 3 (Hp2 – infection after 2 h incubation in PBS), 4–9 (cells infected with *H. pylori*, pretreated with different concentration 4 (13.5 μg/ml), 5 (3.375 μg/ml), 6 (0.422 μg/ml), 7 (0.053 μg/ml), 8 (0.007 μg/ml) were studied to study the influence of AgNPs on *H. pylori*, phospho-CagA (top panel) and CagA (middle panel) levels. GAPDH (lower panel) served as a loading control.

These cell morphological determinations indicated that AgNPs could impact on the *H. pylori* mediated CagA translocation and/or phosphorylation. Therefore, the amount of total CagA and tyrosine-phosphorylated CagA in *H. pylori*-infected AGS cells was investigated by western blotting. Eukaryotic GAPDH was detected as control to ensure equal loading of host cell proteins. The phosphorylation status of CagA (pCagA) was determined using an anti-phosphotyrosine-specific antibody (4G10) (**Figure 7**). While the CagA antibody detects CagA protein in adherent bacteria and in the AGS cell cytoplasm, pCagA is restricted strictly to injected CagA within the host cell cytoplasm. The results of the immunoblotting analysis revealed significant concentrations dependent reduction in the translocation and phosphorylation of CagA after infection of AGS cells with AgNP pretreated *H. pylori*.

**Figure 8:**
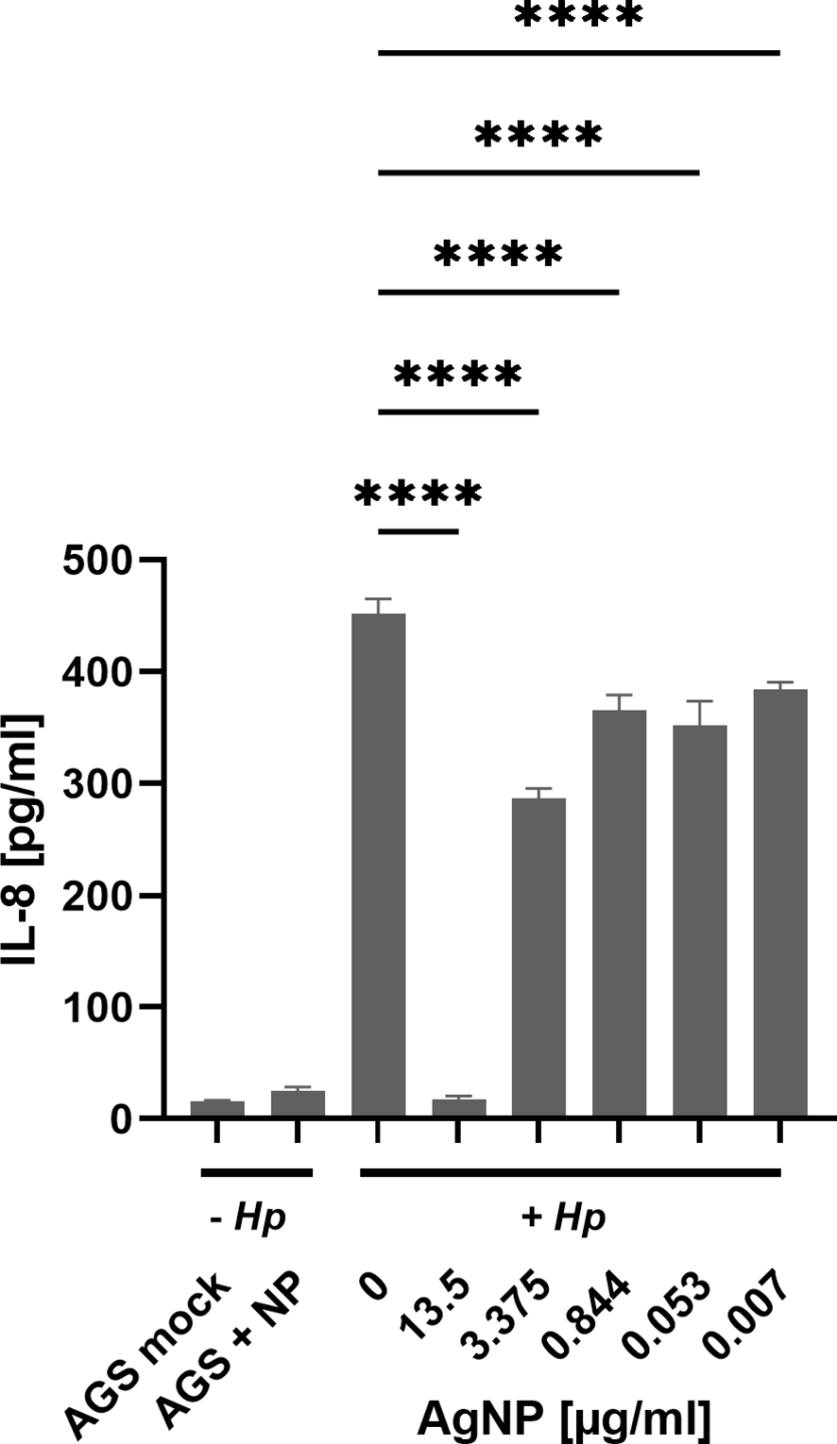
IL-8 expression assessed by ELISA in AGS cells across distinct experimental conditions. Untreated – Hp (AGS mock - cell control, AGS + NP - cells with 13.5 μg/ml AgNPs). Cells treated with *H. pylori*, + Hp (Hp infection after 2 h incubation in PBS (0 μg/ml) and cells infected with *H. pylori* pre-incubated with AgNPs (13.5; 3.375; 0.422; 0.053; 0.007 μg/ml, respectively) in PBS for 2 h). Quantitative data are presented as mean ± standard deviation of interleukin concentration from three independent experiments. **** p≤0.0001.

Our results provide insight into the phosphorylation dynamics of CagA, a key contributor to *H. pylori* pathogenesis (Reyes, 2023). Notably, the incorporation of AgNPs resulted in a significant reduction in both the total amount of CagA as well as diminished levels of phosphorylated CagA. Besides CagA delivery, the type IV secretion system delivers the *H. pylori* metabolite ADP-heptose into host cells, which is an activator of NF-κB mediated IL-8 response (Faass et al., 2021; El Filaly et al., 2023). To address whether AgNPs affect T4SS dependent delivery machinery apart from CagA protein transport, *H. pylori*-induced interleukin-8 (IL-8) secretion, which is dependent on T4SS mediated delivery of ADP-heptose, was analyzed. The levels of IL-8 revealed distinct patterns across these conditions (**Figure 8**). Untreated AGS cells and those treated with AgNPs alone exhibited minimal IL-8 production, contrasting sharply with the robust induction observed in the *H. pylori* infection control group (0 μg/ml AgNPs). Pretreatment of *H. pylori* with various concentrations of AgNPs, resulted in a significant decrease in IL-8 secretion. Of particular note was the pronounced reduction observed at an AgNPs concentration of 13.5 μg/ml, where IL-8 secretion plummeted to 17.4 pg/ml from the level of 452.2 pg/ml, indicative of IL-8 concentration in AGS cells infected with untreated *H. pylori*. Moreover, across all other tested concentrations, a consistent and statistically significant reduction in IL-8 secretion was observed, ranging between 287 pg/ml and 384 pg/ml, with a clear trend towards decreasing concentrations of AgNPs.

## 4 Discussion

The synthesis strategy of AgNPs and thus their specific characteristics play pivotal roles for their intrinsic properties and potential applications. In this study, sodium borohydride served as the reducing agent for the synthesis of small-sized AgNPs. However, dispersions prepared in this manner are often prone to aggregation, especially when in contact with electrolytes (Fernando et al., 2020). To forestall nanoparticle aggregation within the complex bacterial medium, gelatine is a suitable stabilizing agent (Sivera et al., 2012). Characterization techniques including DLS, TEM revealed the presence of small nanoparticles with narrow size distribution, which was together with the stability of AgNPs confirmed also by UV/VIS spectroscopy. This combination of precise size control and effective stabilization emphasizes the suitability of the chosen synthesis approach and provides a solid basis for subsequent investigation of their diverse applications and interactions.

The bactericidal activity of AgNPs has been attributed to their ability to release silver ions, to produce reactive oxygen species and to cause loss of bacterial membrane integrity and subsequent membrane damage (Gurunathan et al., 2018; Jang et al., 2022). Similar mechanisms of action have been also reported for other nanomaterials used in the treatment of *H. pylori* (Au, Bi, Cu, Pd, ZnO) (Asgari et al., 2022; Yin et al., 2023). The antimicrobial potential of the AgNPs synthesised in this study against *H. pylori* was evaluated using real-time growth monitoring and CFU assays. Real-time growth curves revealed a dose-dependent inhibition of *H. pylori* growth, with higher AgNPs concentrations leading to strong growth inhibition. The dose-dependent inhibition of bacterial growth further emphasized the importance of nanoparticle concentration in achieving effective antimicrobial outcomes. Additionally, the real-time monitoring approach employed in this study provided valuable insights into the kinetics of *H. pylori* growth inhibition by AgNPs, offering a comprehensive understanding of their antimicrobial efficacy over time. This enabled identification of effective concentrations for bacterial growth inhibition (MIC 3.375 μg/ml) and revealed time required to exert antimicrobial effect of the nanoparticles, helping to optimise treatment duration and dosage regimens. The MBC was determined to be 3.375 μg/ml (growth curves) and 13.5 μg/ml (CFU assay). The different MBC obtained from the real-time growth and CFU assays arose from the distinct emphases of these methodologies. Growth curves provide a dynamic representation of bacterial growth over time (48 hours), whereas CFU assays provide a quantitative measure of bacterial killing, identifying the concentration at which bacterial survival is significantly reduced within only two hours of exposure time. The MIC values determined in this study are consistent with those reported for silver nanoparticles by several other research groups (Amin et al., 2014; Saravanakumar et al., 2019; Mansouri et al., 2022). The action of AgNPs in the stomach is influenced by their size distribution, the formulation and to a significant extent, gastric pH. Depending on these conditions, not only increased shedding of Ag ions has been reported, but also particle growth or aggregation (Axson et al., 2015). *In vivo*, particles have also been reported to cross gastrointestinal epithelia and to accumulate in the liver (Zhang et al., 2022; Noga et al., 2023). In numerous *in vitro* toxicity studies, high doses of nanoparticles were found to increase reactive oxygen species and nitric oxide levels. Furthermore, AgNPs induce DNA damage and apoptosis (Noga et al., 2023). The observed concentration-dependent reduction in AGS cell viability highlights the importance of carefully selecting nanoparticle concentrations and formulations to ensure both experimental integrity and cell viability. Furthermore, these results provide valuable insights into the biocompatibility of AgNPs with gastric epithelial cells, shedding light on their potential applications in the context of *H. pylori* infection and suggesting avenues for further research. Yet, the complex gastric environment, including gastric juice and chyme, must be taken into account for future studies. The different IC50 values for *H. pylori* and AGS cells to AgNPs in our study, suggest a therapeutic window where AgNPs can effectively inhibit bacterial growth without inducing significant cytotoxic effects on the host. To further widen this window, functionalisation of the silver nanoparticles could enhance the specificity towards bacterial targets while mitigating adverse effects on the host.

Upon infection with *H. pylori*, AGS cells undergo a dramatic change in morphology, characterized by elongation and a migratory phenotype resulting in loss of cell-cell contacts. This elongation process is a well-known consequence of CagA translocation and tyrosine phosphorylation (Moese et al., 2004). Remarkably, pre-incubation of *H. pylori* with AgNPs exerted a strong influence on these morphological changes. The examination of AGS cells after infection with AgNP-pretreated *H. pylori* revealed concentration-dependent effects of AgNPs on cell morphology. Of note, these effects on the delivery of the pathogenicity factor CagA surpass the killing activity observed in cfu assays, indicating, that even sublethal doses of AgNPs are able to block pathogenic processes in *H. pylori*. It has been previously reported, that AgNPs can inhibit enzyme secretion in bacteria (Jahan et al., 2024), and silver nanoparticles have been shown to deregulate genes associated with the type VI secretion system in *Klebsiella pneumoniae* (Hamida et al., 2020). The observed reduction in total CagA signifies a reduction in bacterial viability and thus lower bacterial numbers and/or lower adhesion to host cells, whereas diminished phospho-CagA signal is indicative of impaired injection and/or phosphorylation in the host cells. This suggests that, in addition to the bactericidal activity, AgNPs have a strong potential to modulate CagA translocation and/or phosphorylation, and to specifically block *H. pylori* pathogenicity mechanisms. Future investigations are needed to elucidate the mechanisms by which AgNPs interfere with *H. pylori’s* type IV secretion system (T4SS), disrupt its adhesion to host cells, and modulate host cell kinase signalling pathways.

*H. pylori* infection of AGS cells induces a strong inflammatory response, as evidenced by a strong induction of interleukin-8 secretion (Sansonetti et al., 1999). Similar to the results seen for phospho-CagA, increasing concentration of AgNPs diminished the amounts of secreted IL-8, suggesting a reduction in the pro-inflammatory response to infection. Notably, both phosphorylated CagA and IL-8 were reduced at a concentration of 13.5 μg/ml AgNPs. The observation that the impact of AgNPs on *H. pylori* pathogenicity outperforms the bactericidal activity prompts the speculation that this effect may be caused by predominantly targeting the T4SS machinery. Given the distinct signalling cascades involved in NF-κB signalling and CagA phosphorylation, respectively, we analyzed the CagA independent, but ADP-heptose dependent stimulation of IL-8 secretion (Brandt et al., 2005; Pfannkuch et al., 2019). The highest concentration of AgNPs (13.5 μg/ml) results in a loss of total CagA and IL-8 secretion and suggests bacterial eradication. However, it is notable that IL-8 secretion remains largely unaffected by sublethal doses of AgNPs, where significant loss of CagA injection – as indicated by its phosphorylation – is observed. Direct stimulation of interleukin-8 secretion by AgNPs has been reported in CaCo-2 cells (Polet et al., 2020), however this was not observed in the AGS cell line in our study. This suggests a nuanced interplay between AgNPs and host responses, where the modulation of inflammatory markers like IL-8 may not be as pronounced as expected. These findings highlight the complexity of host-pathogen-nanoparticle dynamics and underscore the need for further investigation to fully elucidate the mechanisms underlying these interactions.

## 5 Conclusion

The present study demonstrates the significant antimicrobial potential of AgNPs against *H. pylori* infection. The AgNPs used were able to target *H. pylori* at a concentration that induced only minor cytotoxic effects in our host cell model. Animal models of *H. pylori* infection show promising first results for the use of AgNPs *in vivo* (Kuo et al., 2014), but failed to achieve complete eradication of the infection. Yet, finetuning the AgNP formulation or functionalization of the AgNP could lead to an even wider therapeutic window and increase the efficacy of AgNP treatments. Given the issues of particle instability in the acidic gastric compartment, combination therapy with proton pump inhibitors may be a promising avenue for future studies. Due to the widespread use of AgNPs in industry the daily consumption of AgNPs by ingestion is estimated around 20–80 µg by the WHO (De Matteis, 2017), therefore short-term treatments with higher doses may impose only minor additional toxicity burden. Nevertheless, the toxicity profile of AgNPs is a major concern and must be critically assessed when considering their use in humans.

## Data availability statement

The original contributions presented in the study are included in the article/**Supplementary Material**. Further inquiries can be directed to the corresponding authors.

## Ethics statement

Ethical approval was not required for the studies on humans in accordance with the local legislation and institutional requirements because only commercially available established cell lines were used. Ethical approval was not required for the studies on animals in accordance with the local legislation and institutional requirements because only commercially available established cell lines were used.

## Author contributions

LH: Conceptualization, Investigation, Methodology, Writing – original draft, Writing – review & editing. GP: Conceptualization, Formal analysis, Methodology, Supervision, Writing – review & editing. SW: Conceptualization, Methodology, Writing – review & editing. LK: Conceptualization, Methodology, Project administration, Writing – review & editing. AP: Writing – review & editing.
