# Supplementary material for: Implications of silver nanoparticles for H. pylori infection: modulation of CagA function and signaling
Source: Front Cell Infect Microbiol. 2024 Jun 25;14:1419568. doi: 10.3389/fcimb.2024.1419568 (PMC11231068; doi:10.3389/fcimb.2024.1419568)
Supplement: Supplementary file 1 [file DataSheet_1.docx]

Supplementary Material

# Supplementary Figures and Tables

## Supplementary Figures


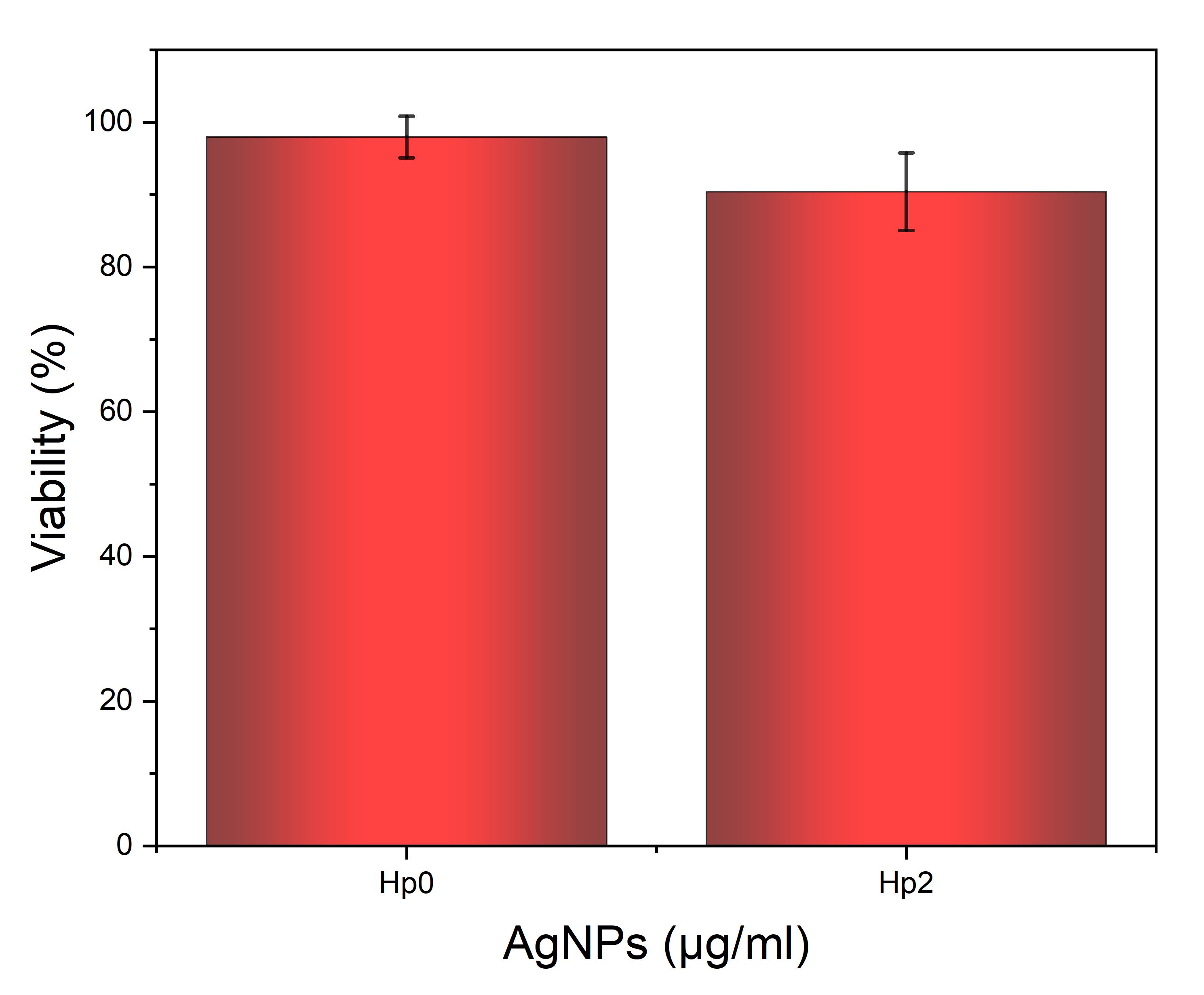


**Supplementary Figure 1.** Cell viability for *H. pylori* controls: Viability of untreated bacteria (Hp0) and *H. pylori* viability after incubation in PBS for two hours (Hp2) under microaerophilic conditions. Quantitative data are presented as mean ± standard deviation of cell viability percentages from three independent experiments. The reference "Hp" signifies the condition without nanoparticles, serving as the non-treatment control and was defined as 100%.
